# Time lag between functional and structural lymphatic changes after lymphadenectomy: Insights from ICG lymphography and lymphatic ultrasound

**DOI:** 10.1371/journal.pone.0345408

**Published:** 2026-03-23

**Authors:** Hisako Hara, Mitsuko Hirai, Makoto Mihara, Takashi Hirayama, Yasuhisa Terao

**Affiliations:** 1 Department of Lymphatic and Reconstructive Surgery, JR Tokyo General Hospital, Tokyo, Japan; 2 Department of Obstetrics and Gynecology, Juntendo University Hospital, Tokyo, Japan; 3 Lymphedema Clinic Tokyo, Tokyo, Japan; Athens Medical Group, Psychiko Clinic, GREECE

## Abstract

Postoperative lower limb lymphedema is a common complication following pelvic or para-aortic lymphadenectomy for gynecologic cancers. Early detection of lymphatic dysfunction is crucial, but the temporal relationship between functional and structural changes remains unclear. This prospective observational study aimed to compare indocyanine green (ICG) lymphography and lymphatic ultrasound findings at multiple time points in the early postoperative phase. We enrolled 23 patients (46 lower limbs) who underwent pelvic and/or para-aortic lymphadenectomy for gynecologic malignancies. Each patient underwent ICG lymphography and lymphatic ultrasound preoperatively and at 1, 3, and 9 months postoperatively. ICG patterns were categorized as linear, splash, or stardust, while lymphatic vessel dilation ≥0.3 mm was defined as abnormal on ultrasound. At least one abnormal ICG finding was observed in 52.2% of limbs, and abnormal ultrasound findings were present in 65.2%. Among limb-timepoints with abnormal ICG findings, lymphatic dilation on ultrasound was observed in 32.6% overall, increasing to 52.6% at 9 months postoperatively, indicating increasing concordance between functional and structural abnormalities over time. Splash patterns on ICG were often not accompanied by lymphatic dilation, whereas stardust patterns were more likely to coincide with structural changes, especially at 9 months. Skin thickness increased significantly in the medial and lateral lower leg regions in limbs with stardust patterns. Our findings demonstrate a temporal dissociation between functional abnormalities detected by ICG and structural changes detected by ultrasound, suggesting that ICG lymphography may be more sensitive in the early phase. The combined use of both modalities may help capture the continuum from early functional disturbance to later structural remodeling and inform the optimal timing of intervention.

## Introduction

Surgical treatment for gynecologic malignancies, particularly pelvic and para-aortic lymphadenectomy, is effective in controlling cancer progression. However, it carries the risk of postoperative complications, among which lower extremity lymphedema is one of the most significant. Lymphedema, if left untreated, can lead to chronic swelling, pain, skin sclerosis, and recurrent cellulitis, resulting in considerable physical and psychological distress and a reduction in quality of life. The incidence of lower limb lymphedema following gynecologic cancer surgery is estimated to be 10–30% [1,2]. In recent years, efforts to reduce the incidence of lymphedema, such as robotic surgery and sentinel lymph node biopsy, have been increasingly adopted. Nevertheless, early diagnosis of lymphedema remains a clinical challenge.

Current diagnostic modalities for lymphedema include patient history, physical examination, limb circumference measurements, indocyanine green (ICG) lymphography, and lymphoscintigraphy. Among these, ICG lymphography is a functional imaging technique that allows real-time visualization of lymphatic flow following subcutaneous injection of ICG [3–7]. Specific dermal backflow patterns, such as the “splash” and “stardust” patterns, are considered indicative of lymphatic dysfunction due to valve incompetence or increased intraluminal pressure, and are widely utilized in clinical practice.

Lymphatic ultrasound, a more recent and promising modality, uses high-frequency ultrasound probes to noninvasively visualize collecting lymphatic vessels within the subcutaneous fat layer [8,9]. While ultra-high frequency probes enable the evaluation of superficial lymphatics located within 5 mm of the skin surface, collecting lymphatic vessels in patients with lymphedema are typically found at depths of approximately 1 cm, making them accessible using standard high-frequency probes available in most medical institutions [10,11]. Lymphatic vessels in lymphedema are known to undergo characteristic morphological changes—ranging from normal to dilated, contracted, and sclerotic types—which we have previously classified as the NECST classification [12,13]. The NECST classification describes the pathological progression of lymphatic vessel degeneration in lymphedema, based on both histopathological findings (light and electron microscopy) and intraoperative microscopic observations of lymphatic morphology. Lymphatic ultrasound enables the visualization of these degenerative changes and has been increasingly utilized for both diagnosis and preoperative planning for lymphaticovenous anastomosis (LVA) surgery [14–16]. In our previous work, we demonstrated that a lymphatic vessel diameter exceeding 0.3 mm serves as a structural marker of lymphatic dysfunction and may aid in the diagnosis of lymphedema [9]. Moreover, we have shown that the diagnostic sensitivity of lymphatic ultrasound in patients with chronic-stage lymphedema is comparable to that of ICG lymphography [8].

Despite these advancements, no studies to date have explored the temporal relationship between ICG lymphography and lymphatic ultrasound findings in the early postoperative period. The present study aims to address this gap by evaluating both modalities at multiple time points following gynecologic cancer surgery. The objective of this study was to elucidate the respective roles of ICG lymphography and lymphatic ultrasound in assessing lymphatic function and structure over time in postoperative patients. We hypothesized that functional abnormalities in the lymphatic system would become detectable earlier by one imaging modality than by the other, resulting in a temporal dissociation between functional and structural assessments in the early postoperative period.

## Patients and methods

This prospective observational study enrolled patients who underwent pelvic and/or para-aortic lymphadenectomy for gynecologic malignancies between 1 November 2020 and 31 December 2023. Eligible participants were women aged 20 years or older scheduled to undergo surgery for cervical or ovarian cancer involving pelvic lymph node dissection. Additional inclusion criteria included an Eastern Cooperative Oncology Group (ECOG) performance status of 0–2 and adequate bone marrow, hepatic, and renal function. Exclusion criteria were: prior pelvic or abdominal radiotherapy, history of chemotherapy, known hypersensitivity to indocyanine green (ICG), or a history of iodine allergy. Patients who developed iodine allergy postoperatively were excluded because further ICG lymphography could not be safely performed during follow-up, making longitudinal comparison impossible.

Of the 30 patients initially enrolled, 7 were excluded: 3 withdrew consent, 2 were unable to continue due to difficulties with outpatient visits during the COVID-19 pandemic, 1 underwent a change in surgical procedure, and 1 developed an iodine allergy postoperatively. Consequently, data from 23 patients (46 lower limbs) who completed follow-up assessments through 9 months postoperatively were included in the analysis.

All participants were female, with a mean age of 51.7 years (range: 30–70 years). Diagnoses included cervical cancer (n = 11), endometrial cancer (n = 10), ovarian cancer (n = 1), and vaginal cancer (n = 1). All patients underwent pelvic and/or para-aortic lymphadenectomy, with a mean of 45.5 lymph nodes (range: 14–142) resected. Chemotherapy with taxanes was administered to 8 patients (34.8%), non-taxane chemotherapy to another 8 patients (34.8%), and radiation therapy to 9 patients (39.1%). The mean body mass index (BMI) was 23.0 kg/m² (range: 17.7–35.9).

All patients underwent preoperative assessments, including ICG lymphography and lymphatic ultrasound, between the decision for surgery and the scheduled operation. These same evaluations were repeated postoperatively at 1, 3, and 9 months, totaling four time points. All examinations were performed by a single physician experienced in lymphedema diagnosis and management (HH), and findings were verified by a second experienced physician (MM).

**Fig 1:**
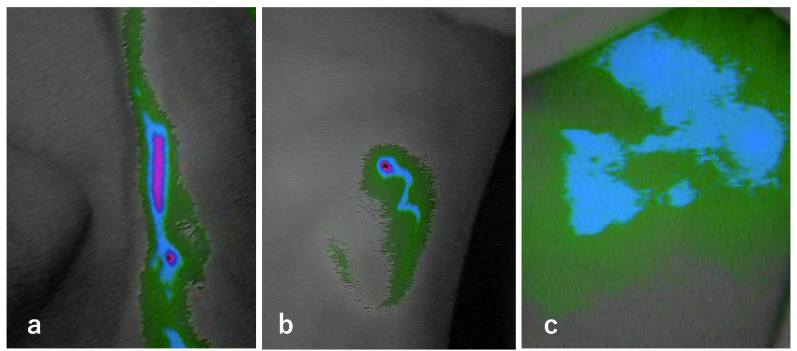
ICG lymphography findings. The diffuse pattern, which represents severe abnormality, was not observed in this study. **(a)** Linear pattern, indicating normal lymphatic flow. **(b)** Splash pattern, an early abnormal finding resembling brush strokes. (c) Stardust pattern, indicating moderate abnormality.

For ICG lymphography, approximately 0.05 mL of ICG was injected subcutaneously at three sites on each lower limb: the first interdigital space, lateral malleolus, and lateral midline at the superior patellar level. After a 2-hour interval during which patients were free to walk or rest in a café, lymphatic flow was assessed using a near-infrared camera (Photodynamic Eye; Hamamatsu Photonics, Hamamatsu, Japan). ICG patterns were classified into four categories: linear (normal), splash, stardust, and diffuse (abnormal) [17], and findings were recorded for each limb (Fig 1).

**Fig 2:**
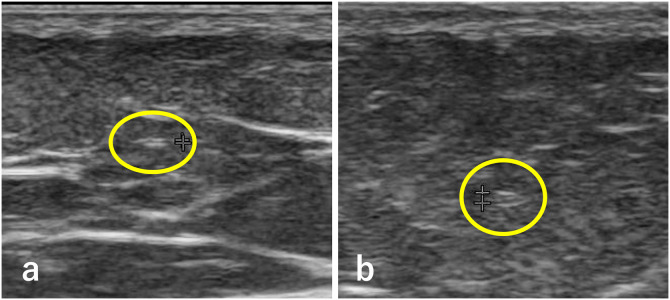
Lymphatic ultrasound findings. Yellow circles indicate the lymphatic vessels. **(a)** Normal lymphatic vessel without dilation, appearing as a high-echo structure resembling an equal sign. **(b)** Dilated lymphatic vessel due to lymphatic stasis.

On the same day, lymphatic ultrasound was performed using the Noblus ultrasound system equipped with an 18-MHz linear probe (Hitachi Medical Corp., Tokyo, Japan). Each limb was divided into four regions: thigh and lower leg, medial and lateral aspects. Lymphatic vessels were identified using previously reported D-CUPS (Doppler, Crossing, Uncollapsible, Parallel, and Superficial fascia) criteria to distinguish them from veins [8]. The most clearly visualized lymphatic vessel in each region was evaluated in cross-section, and its internal diameter was measured using the system’s caliper function. Based on our prior study [9], lymphatic vessels with a diameter ≥0.3 mm were considered abnormal, and categorized into dilated, sclerotic, or obstructed types (Fig 2). This cutoff was selected because a lymphatic vessel diameter of ≥0.3 mm is considered to represent clinically meaningful dilation and provides a practical threshold for identifying structural lymphatic changes in routine clinical practice. A limb was classified as having abnormal lymphatics if at least one abnormal vessel was identified.

Additionally, skin thickness was measured in each of the four regions using the ultrasound system’s caliper function.

To explore the relationship between functional abnormalities on ICG lymphography and structural changes on lymphatic ultrasound, we examined the temporal concordance and discordance between the two modalities across all limb-timepoints. To evaluate temporal trends, the number of limbs with and without lymphatic dilation was assessed at each time point, and patterns of concordance or discordance between ICG and ultrasound findings over time were analyzed. Both limbs from the same patient were analyzed as independent units. Because of the limited sample size and the exploratory design of this study, no statistical adjustment for within-patient correlation was performed.

This study was approved by the institutional ethics committee (Approval number: J20-008). Written informed consent was obtained from all participants prior to enrollment.

## Results

No adverse events associated with ICG lymphography or lymphatic ultrasound were observed. Abnormal findings on ICG lymphography (either splash or stardust pattern) were observed at least once during the study period in 24 of 46 limbs (52.2%). Lymphatic ultrasound revealed abnormal findings in 30 of 46 limbs (65.2%).

**Fig 3:**
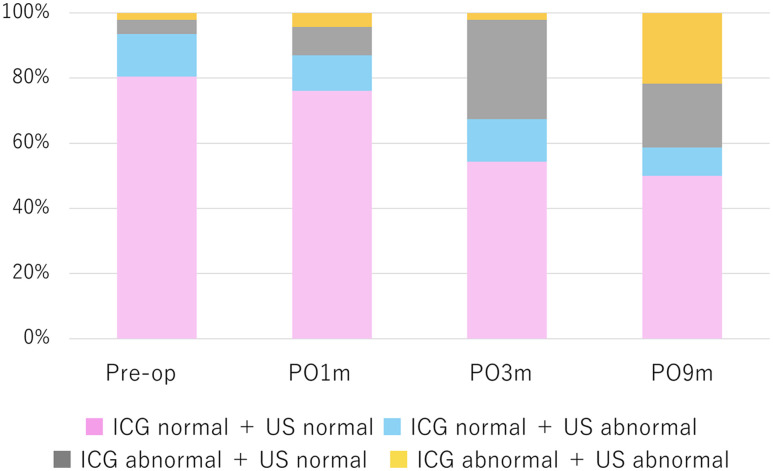
Temporal changes in concordance and discordance between indocyanine green (ICG) lymphography and lymphatic ultrasound across postoperative timepoints. The stacked bars represent the proportions of limb-timepoints classified into four categories according to the agreement between the two imaging modalities: (1) Concordant abnormal findings — abnormal ICG findings with lymphatic dilation on ultrasound; (2) Discordant findings indicating functional abnormality without structural change — abnormal ICG findings without lymphatic dilation on ultrasound; (3) Discordant findings indicating structural change without functional abnormality — normal ICG findings with lymphatic dilation on ultrasound; and (4) Concordant normal findings — normal findings on both ICG lymphography and lymphatic ultrasound. Overall, the figure shows a temporal shift from predominantly discordant findings in the early postoperative phase to an increased proportion of concordant abnormal findings at 9 months.]US: ultrasound.

A total of 184 limb-timepoints were analyzed based on the four assessments conducted on 46 limbs. Among the 43 limb-timepoints that showed abnormal ICG findings, 14 (32.6%) also demonstrated lymphatic dilation on ultrasound. In contrast, of the 141 limb-timepoints without abnormal ICG findings, 21 (14.9%) exhibited lymphatic dilation on ultrasound. To characterize the temporal relationship between functional abnormalities on ICG lymphography and structural changes on lymphatic ultrasound, we examined the concordance and discordance between the two modalities across all limb-timepoints (Fig 3). In the early postoperative phase, discordant findings were common, with abnormal ICG findings frequently observed in the absence of lymphatic dilation on ultrasound, indicating functional abnormalities without overt structural change. At 3 months postoperatively, the proportion of discordant findings reflecting functional abnormalities without structural change increased, whereas concordant abnormal findings remained limited. By 9 months postoperatively, the proportion of concordant abnormal findings increased markedly, suggesting progressive structural remodeling following earlier functional impairment.

**Fig 4:**
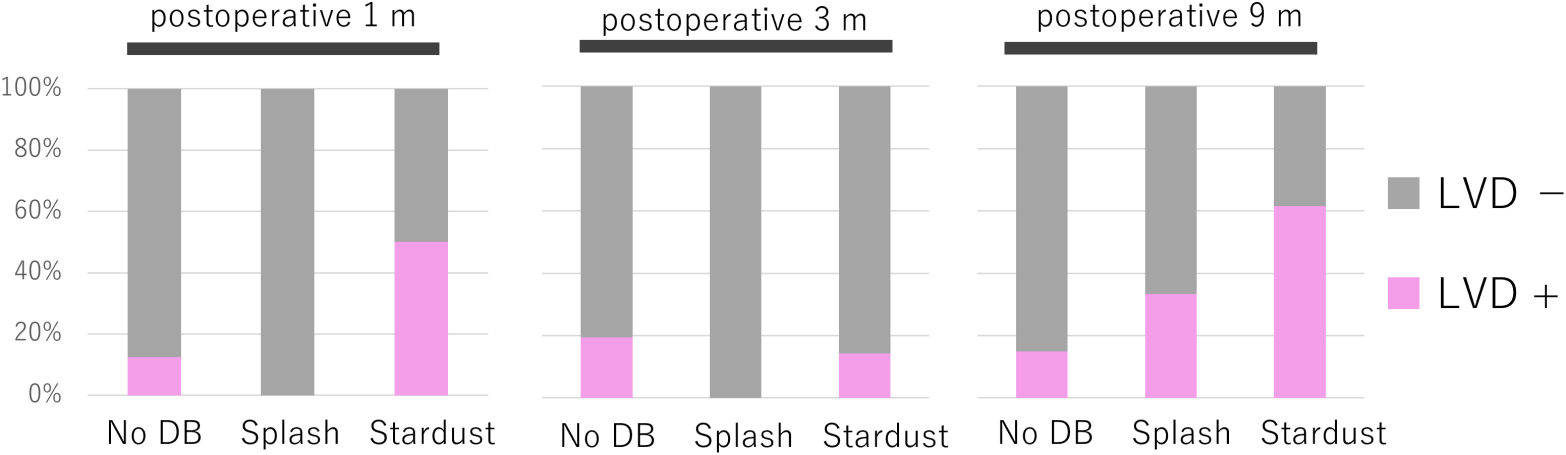
Association between indocyanine green (ICG) lymphography patterns and lymphatic dilation on ultrasound over time. This figure shows the proportion of limbs with lymphatic vessel dilation (LVD) according to ICG lymphography patterns (normal, splash, and stardust) at each postoperative time point. Splash patterns were frequently observed without accompanying lymphatic dilation in the early postoperative phase, whereas stardust patterns were more consistently associated with structural changes, particularly at 9 months postoperatively, highlighting the temporal progression from functional to structural abnormalities. US: ultrasound, DB: dermal backflow.

To further investigate the relationship between ICG findings and lymphatic ultrasound, the ICG findings were categorized as normal, splash, or stardust, and their association with lymphatic dilation was examined at each timepoint (Fig 4). At 1 month postoperatively, stardust patterns were observed in four limbs. These findings were distributed in the medial thigh in two limbs and in the medial lower leg in two limbs, indicating that early postoperative stardust changes were not confined to the inguinal region but also extended distally. Among limbs exhibiting splash patterns, no lymphatic dilation was observed at 1 or 3 months, with dilation identified in only 33.3% of cases by 9 months. In contrast, limbs with stardust patterns more frequently demonstrated lymphatic dilation throughout the study, with a particularly high rate of 61.5% at 9 months postoperatively.

**Fig 5:**
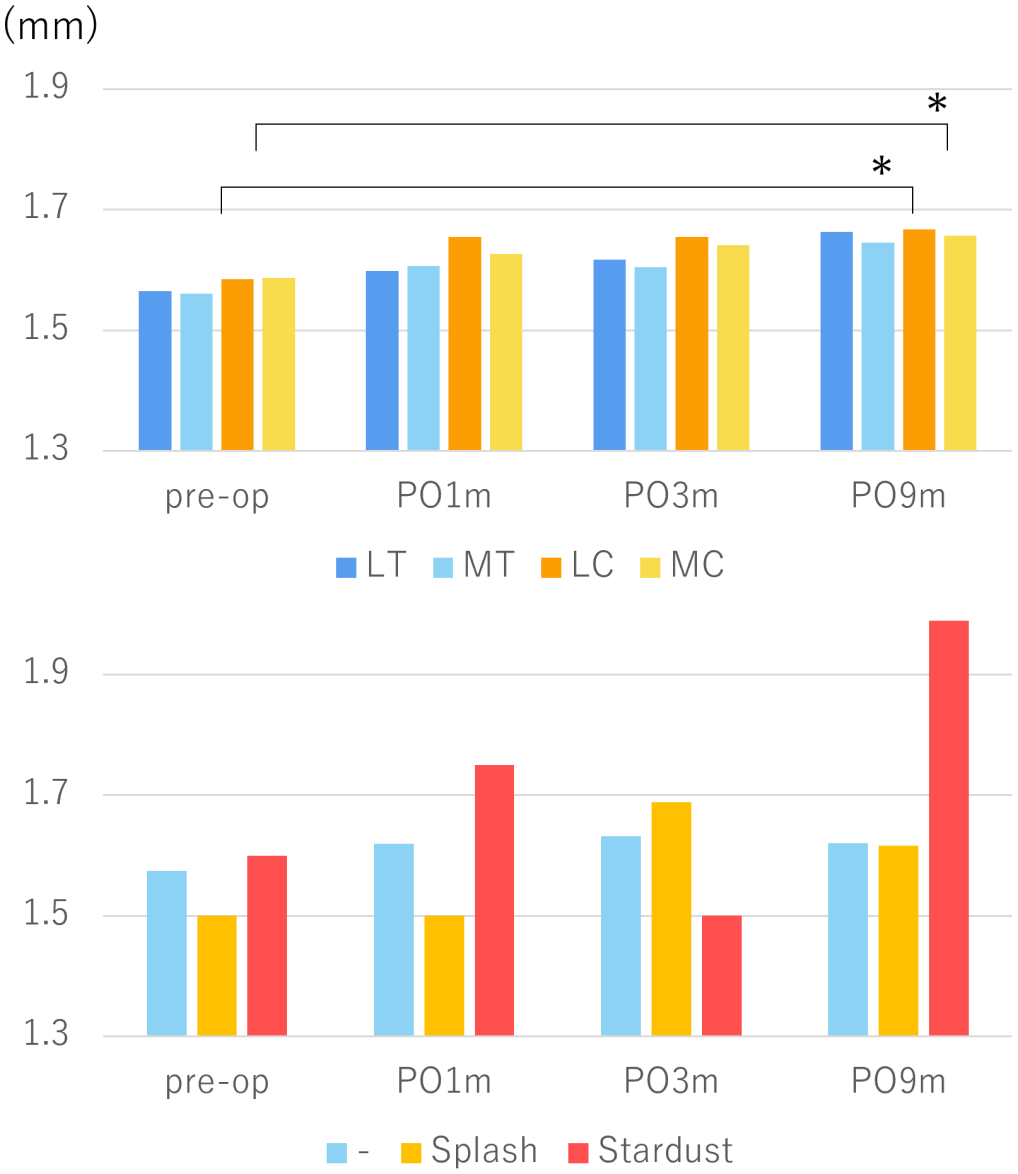
Temporal changes in skin thickness after gynecologic cancer surgery. Skin thickness significantly increased at 9 months in the medial and lateral lower legs (p = 0.035 and p = 0.020). Thickness increased steadily in the stardust group, peaked transiently in the Splash group and Stardust group, and remained stable in the no abnormality group (-). LT; lateral thigh, MT; medial thigh, LC; lateral calf, MC; medial calf.

Skin thickness showed a gradual increase over time (Fig 5), particularly in the medial and lateral lower leg regions, where statistically significant increases were observed at 9 months compared to baseline (p = 0.035 and p = 0.020, respectively). When stratified by ICG pattern, the stardust group demonstrated a marked increase in skin thickness, reaching 2.0 mm by 9 months despite instability in earlier phases. The splash group showed a transient increase between 1 and 3 months postoperatively, followed by a slight decrease at 9 months. Limbs without ICG abnormalities showed stable skin thickness throughout the 9-month observation period.

## Discussions

This is the first study to longitudinally evaluate lymphatic flow disturbance using both indocyanine green (ICG) lymphography and lymphatic ultrasound in patients after gynecologic cancer surgery. Our findings demonstrate that functional abnormalities—reflected as dermal backflow on ICG lymphography—tend to precede structural changes such as lymphatic vessel dilation detectable on ultrasound. This highlights the distinct phases of disease progression captured by each modality and provides new insights into the appropriate timing of diagnosis and therapeutic intervention. Neither ICG lymphography nor lymphatic ultrasound can be considered a gold standard for early lymphatic dysfunction; therefore, this study focuses on the temporal relationship between functional and structural findings rather than diagnostic performance.

This study does not address the indications for lymphadenectomy or compare different nodal management strategies. Rather, it focuses on postoperative lymphatic functional and structural changes after surgical lymphatic disruption. Because all patients underwent systematic lymphadenectomy, the findings primarily reflect the effects of extensive lymphatic injury and may not be generalizable to patients undergoing SLN-based staging.

Lymphatic dilation on ultrasound was often absent when dermal backflow was already evident on ICG lymphography, indicating that functional disturbance may precede detectable structural remodeling. Over time, concordance between ICG and ultrasound findings increased, reaching its highest level at 9 months postoperatively, supporting a model in which functional impairment precedes anatomical change.

In a small number of cases, lymphatic dilation was observed on ultrasound despite normal ICG findings. This discrepancy may reflect transient, reversible dilation rather than irreversible remodeling, consistent with our previous intraoperative study showing that more than 60% of lymphatic vessels exhibited dilation or sclerosis even in areas with normal ICG patterns [13]. The transient fluctuation in lymphatic dilation observed between 1 and 3 months postoperatively may reflect dynamic changes in lymphatic load rather than true hyperfunction. Immediately after lymphadenectomy, the remaining lymphatics are likely exposed to increased functional burden, resulting in temporary dilation. In some limbs, dilation decreased by 3 months, possibly due to adaptive remodeling or compensatory lymphangiogenesis, whereas in others, dilation progressed again by 9 months, suggesting insufficient compensation and subsequent structural deterioration. Because this study focused on longitudinal imaging patterns rather than quantitative functional assessment, dynamic parameters such as flow velocity were not systematically analyzed; therefore, these interpretations remain speculative. Compared with our previous study in chronic lymphedema, in which lymphatic ultrasound showed a high level of concordance with ICG findings (95%) because of advanced structural changes, the present early postoperative cohort demonstrated substantially lower concordance (32.6%). This pattern is consistent with what we refer to as a *“*functional-only” phase, in which functional abnormalities detected by ICG lymphography may precede detectable structural changes on ultrasound. Concordance increased over time, reaching 52.6% at 9 months postoperatively, while the proportion of limbs with normal ultrasound findings remained consistently high throughout the study period. These observations led us to propose the concept of a “functional-only” phase in the early postoperative period, in which functional abnormalities detected by ICG lymphography may precede detectable structural changes on ultrasound. The notion of a “functional-only” phase is derived from the observed imaging patterns in this cohort and should be interpreted as a conceptual framework rather than a definitive clinical stage.

Akita et al. previously reported that, in patients after gynecologic cancer surgery, the presence of a splash pattern on ICG lymphography was significantly associated with the subsequent development of clinical lymphedema (relative risk 1.62), and suggested that splash may serve as an early warning sign [18,19]. In our study, splash patterns emerged early and frequently progressed to stardust patterns over time, consistent with Akita’s findings. Their study also pointed out that timely therapeutic intervention (e.g., compression therapy) at the stage of splash patterns is challenging. Our present study builds on this work by identifying the lag between functional and structural changes, providing evidence to guide the timing of interventions more precisely. However, the clinical significance of these early functional abnormalities detected by ICG lymphography, particularly splash patterns, remains uncertain, as they were not correlated with clinical lymphedema or patient-reported outcomes in the present study. Accordingly, these findings should be interpreted as hypothesis-generating, and further studies are needed to clarify their prognostic and clinical relevance.

We observed that the severity of ICG abnormalities was associated with increases in skin thickness over time. Limbs with stardust patterns showed progressive skin thickening, whereas splash patterns were generally accompanied by minimal or reversible changes. Similar to lymphatic dilation, increases in skin thickness lagged behind ICG abnormalities, with no significant thickening observed until after 3 months postoperatively. This delayed pattern is consistent with previous reports in chronic lymphedema, in which skin thickening becomes more prominent in later stages [20].

These findings have potential implications for clinical decision-making in the early postoperative period. When abnormal ICG findings are present without structural changes on ultrasound, clinicians face uncertainty regarding the timing of intervention. Early treatment based solely on splash patterns may risk overtreatment, whereas delaying intervention until structural changes appear may miss a window to prevent irreversible remodeling. Further long-term studies are needed to clarify the optimal timing of intervention.

This study was not designed to establish diagnostic criteria or severity grading for clinical lymphedema. Instead, our objective was to investigate early lymphatic alterations detectable by imaging modalities before the development of overt clinical signs and symptoms. In this context, the observed differences between ICG lymphography and lymphatic ultrasound may reflect temporal discordance between functional and structural changes in the postoperative lymphatic system rather than differences in diagnostic performance.

This study has several limitations. First, the observation period was limited to 9 months postoperatively, and the relationship between long-term lymphatic structural changes and the eventual development of clinical lymphedema remains unclear. Given the notable increase in abnormal ultrasound findings at 9 months, extended follow-up is necessary to determine the time point at which the diagnostic sensitivity of lymphatic ultrasound may approach that of ICG lymphography. Second, although the use of a single experienced examiner ensured consistency in image interpretation, it may have limited the reproducibility and external validity of our findings. Future studies involving multiple observers will be essential to assess inter-rater reliability and validate the robustness of imaging interpretation. In addition, the relatively small sample size may limit the generalizability of our findings. Therefore, larger multicenter studies with longer follow-up are warranted.

In conclusion, this study demonstrated that functional abnormalities detected by ICG lymphography appear earlier than structural changes identified by lymphatic ultrasound after gynecologic cancer surgery. Because these two modalities assess different aspects of lymphatic pathology, ICG lymphography is particularly informative in the early postoperative phase, whereas lymphatic ultrasound is highly valuable in the chronic phase for evaluating structural progression. In clinical practice, their combined use may support more appropriate postoperative surveillance by identifying patients who require closer monitoring while avoiding premature intervention in those without emerging structural changes.

## Supporting information

S1 File Raw dataset of lymphatic ultrasound findings in the study cohort. (XLSX)
